# Correction

**DOI:** 10.1002/ehf2.14356

**Published:** 2023-03-20

In the paper by Baratto *et al*.,^1^ Claudia Baratto have two affiliations in the published version.

The correct affiliation should only be:

Department of Cardiovascular, Neural and Metabolic Sciences, Istituto Auxologico Italiano IRCCS, Ospedale San Luca, Milano, Italy

## References

[1] Baratto C , Caravita S , Soranna D , Dewachter C , Bondue A , Zambon A , Badano LP , Parati G , Vachiéry JL . Exercise haemodynamics in heart failure with preserved ejection fraction: a systematic review and meta‐analysis. ESC Heart Fail. 2022; 9: 3079–3091. 35748109 10.1002/ehf2.13979 PMC9715813

